# Role of NLRP3 inflammasome in diabetes and COVID-19 role of NLRP3 inflammasome in the pathogenesis and treatment of COVID-19 and diabetes NLRP3 inflammasome in diabetes and COVID-19 intervention

**DOI:** 10.3389/fimmu.2023.1203389

**Published:** 2023-10-05

**Authors:** Jiayu Zhang, Xuejing Ma, Fuwei Liu, Deju Zhang, Jitao Ling, Zicheng Zhu, Yixuan Chen, Pingping Yang, Yanlin Yang, Xiao Liu, Jing Zhang, Jianping Liu, Peng Yu

**Affiliations:** ^1^ Department of Metabolism and Endocrinology, The Second Affiliated Hospital of Nanchang University, Nanchang, China; ^2^ Huankui Academy, Nanchang University, Jiangxi, Nanchang, China; ^3^ Xiangya School of Medicine, Central South University, Changsha, China; ^4^ Department of Cardiology, The Affiliated Ganzhou Hospital of Nanchang University, Jiangxi, China; ^5^ Food and Nutritional Sciences, School of Biological Sciences, The University of Hong Kong, Hong Kong, Hong Kong SAR, China; ^6^ Department of Anesthesiology, The Second Affiliated Hospital of Nanchang University, Nanchang, China; ^7^ Department of Cardiology, Sun Yat-sen Memorial Hospital, Sun Yat-sen University, Guangzhou, China

**Keywords:** NLRP3, COVID-19, SARS-CoV-2, diabetes, cytokine storm

## Abstract

2019 Coronavirus Disease (COVID-19) is a global pandemic caused by severe acute respiratory syndrome coronavirus-2 (SARS-CoV-2). A “cytokine storm”, i.e., elevated levels of pro-inflammatory cytokines in the bloodstream, has been observed in severe cases of COVID-19. Normally, activation of the nucleotide-binding oligomeric domain-like receptor containing pyrin domain 3 (NLRP3) inflammatory vesicles induces cytokine production as an inflammatory response to viral infection. Recent studies have found an increased severity of necrobiosis infection in diabetic patients, and data from several countries have shown higher morbidity and mortality of necrobiosis in people with chronic metabolic diseases such as diabetes. In addition, COVID-19 may also predispose infected individuals to hyperglycemia. Therefore, in this review, we explore the potential relationship between NLRP3 inflammatory vesicles in diabetes and COVID-19. In contrast, we review the cellular/molecular mechanisms by which SARS-CoV-2 infection activates NLRP3 inflammatory vesicles. Finally, we propose several promising targeted NLRP3 inflammatory vesicle inhibitors with the aim of providing a basis for NLRP3-targeted drugs in diabetes combined with noncoronary pneumonia in the clinical management of patients.

## Highlights

- Activation of the NLRP3 inflammasome affects the cellular response to insulin.
- Activation of NLRP3 inflammasome plays an important role in maintaining endothelial cell function in COVID-19.
- Anti-diabetic drugs can affect the activation of NLRP3 inflammasome.
- NLRP3 can cause a chronic low inflammatory state in diabetic patients.
- Antidiabetic drugs can be used as an adjuvant treatment for COVID-19 patients with diabetes.

## 1 Introduction

Severe acute respiratory syndrome coronavirus 2 (SARS-CoV-2), a member of the β coronavirus family, is the pathogen behind the current 2019 Coronavirus Disease (COVID-19) pandemic, belongs to the genus Coronaviruses in the family Coronaviridae (1). The SARS-CoV-2 virus is transmitted from person to person through respiratory droplets and aerosols. Once inside the human body, the virus binds to host receptors and enters host cells through endocytosis or membrane fusion. The harm of SARS-CoV-2 to the human body is great (2). Common pneumonia caused by SARS-CoV-2 can cause respiratory failure and even multiple organ failure death in severe cases.

Many risk factors may lead to a poor prognosis of COVID-19, such as smoking, diabetes, and obesity. As one of the factors leading to poor prognosis of COVID-19, diabetes can have a detrimental effect on the host’s immunity. In a study of COVID-19 patients in China, the survival rate of non-diabetic COVID-19 patients was much higher than that of diabetic COVID-19 patients (3). Chronic hyperglycemia impairs innate and humoral immunity. In addition, diabetes is associated with a low-grade chronic inflammatory state that promotes the development of inflammation and is therefore more prone to acute respiratory distress syndrome.

Nucleotide-binding oligomeric domain-like receptor containing pyrin domain 3(NLRP3) is a family of intracellular innate immune receptors, one of the most characterized members of the NOD-like receptor family. It is a multiprotein complex in macrophages, dendritic cells, and other non-immune cells, which is composed of a sensor (NLRP3), an adaptor (ASC; Also known as PYCARD), and an effector (caspase 1) composition. As a key component of the innate immune system, NLRP3 inflammasome plays an important role in host defense against bacteria, fungi, and viruses. NLRP3 is also involved in metabolism and inflammation, such as gout, diabetes, insulin resistance, and obesity (4). Existing studies have shown that NLRP3 plays an important role in the occurrence and development of variety infectious diseases such as SARS, MERS, COVID-19, and other coronaviruses. The NLRP3 inflammasome plays a pivotal role in fundamental bodily functions, including immunity and metabolism, by undergoing activation. In general, NLRP3 activation can be divided into two steps, startup, and activation. In the first step of initiation, ice sheet associated molecular patterns (PAMPs) or damage associated molecular patterns (DAMPs) are recognized by toll-like receptors, leading to NF-κB-mediated signaling activation and upregulated transcription of inflammatory-associated components. The second step is activation, in which ATP, disease RNA and other substances are triggered to complete the assembly of the NLRP3, ASC and pre-caspase-1 complex. In severe cases, levels of proinflammatory cytokines rise in the bloodstream, creating a so-called “cytokine storm”. At the same time, many literatures have reported that diabetes with coronavirus is more prone to the release of inflammatory factors, especially IL-6 and IL-18, which are important inflammatory factors downstream of NLRP3 (5). In this context, a better understanding of the activation of NLRP3 inflammasome in diabetes mellitus with COVID-19 will facilitate further research and better treatment of diabetes mellitus with COVID-19. Given the important involvement of NLRP3 inflammasome in COVID-19, this article reviews the possible pathways of NLRP3 activation in diabetes mellitus complicated with COVID-19, the pathogenesis, and the intervention drugs and therapeutic methods based on inhibiting NLRP3 inflammasome in diabetes mellitus complicated with COVID-19 (6).

## 2 Association of NLRP3 activation, SARS-CoV-2 infection, and obesity

### 2.1 SARS-CoV-2 infection and NLRP3

The genome of SARS-CoV-2 has two large open reading frames: ORF1a and ORF1ab. The 5 ‘end of the SARS-CoV-2 genome encodes a polyprotein, which is processed into 16 non-structural proteins by the proteolytic enzyme Mpro (3CLpro) and papain-like protease PLpro 2. Its 3 ‘end encodes an accessory protein and four structural proteins, including spike protein (S), membrane protein (M), envelope protein (E),and nucleocapsid protein (N), which are closely related to the activation of NLRP3 inflammasome (7).

The nucleocapsid protein, or N protein, directly binds to the NLRP3 protein. This binding enhances the association of NLRP3 with ASC, fostering the assembly of the NLRP3 inflammasome. An interesting cascade involving the N protein-MBL-MASP2 axis may also interface with the NLRP3 inflammasome through the dynamic roles of C3a, C5a, and MAC, further activating the inflammasome. As a component of the viral envelope, the SARS-CoV-2 N protein exits the cytoplasm prior to the virus’s assembly, which potentially induces NLRP3 activation, escalating the possibility of an acute inflammatory response (8). Followed by the envelope protein, SARS-CoV-2 E protein, which depends on ROS and K+ extravasation, may initially suppress the host NLRP3 inflammasome response to viral RNA, while possibly increasing the NLRP3 inflammasome response later in infection. E protein inhibits inflammasome priming and NLRP3 inflammasome activation in cultured macrophages. Finally, the spike protein, ACE2 receptor interaction with SARS-CoV-2 spike protein will stimulate the NLRP3 inflammasome, if overactivated, may lead to pyroptosis. Activation of the renin-angiotensin-aldosterone system (RAAS) leads to elevated levels of angiotensin II. The interaction of the angiotensin-converting enzyme 2 (ACE2) receptor with the SARS-Cov-2 spike (S) protein reduces the degradation of Ang II, leading to Ang II accumulation and then activation of the NLRP3 inflammasome. At the same time, over-activating, it may lead to pyroptosis (9).

Meanwhile, the open reading frame of SARS-CoV-2 can also play a role in activating the NLRP3 inflammasome. The SARS-CoV-2 viral porin encoded by ORF3a triggers the NLRP3 inflammatory pathway. The SARS-CoV-2 ORF3a viroporins activate the NLRP3 inflammasome, the most heterogeneous of known inflammasomes. Ectopically expressed ORF3a triggers IL-1β expression via NF-κB, which initiates the inflammasome. ORF3a activates NLRP3 inflammasomes in both ASC-dependent and independent modes. This inflammasome activation requires potassium efflux and oligomerization between the kinases NEK7 and NLRP3 (10).

Nonstructural proteins of SARS-CoV-2 can also affect NLRP3 activation. Two nonstructural proteins (NSPS), NSP1 and NSP13, have been found to inhibit caspase-1-mediated IL-1β activation. SARS-CoV-2 nonstructural protein 6 triggers NLRP3-dependent pyroptosis by targeting ATP6AP1 (11).

Furthermore, the activation of the NLRP3 inflammasome is pivotal in the innate immune response to viral pathogens in humans. The so-called ‘cytokine storm’, stemming from rampant inflammation and unregulated cytokine release, is a prime suspect for the detrimental clinical outcomes seen in COVID-19. The NLRP3 inflammasome is also flagged as a potential interactor with the myeloid differentiation primary response (MYD88). Elevated IL-1β levels, activated TLR4, and the MYD88 pathway converge, stimulating NLRP3 inflammasome and exacerbating SARS-CoV-2 infections. Recent studies revealed heightened levels of inflammasome-associated products, IL-1β, IL-18, and LDH in COVID-19 patients, highlighting the inflammasome’s association with the disease (12). Clinical studies further affirm the crucial role of the inflammasome in moderate to severe SARS-CoV-2 infections.

### 2.2 Activation of NLRP3

Inflammatory bodies are polymorphic complexes formed by pattern recognition receptors activated by various physiological or pathogenic stimuli. They are an important part of innate immune response and can clear pathogens and damage cells. The nucleotide-binding domain leucine-rich receptor (NLR) is a family of intracellular innate immune receptors. One of the most characteristic members of the subfamily is NLRP3, which includes the sensor molecule NLRP3, a spot-like protein associated with apoptosis. Contains the caspase recruitment domain (CARD) and pro-caspase-1 (13).

Activation of the inflammasome is one of the important defense mechanisms in the early stage of infection. Most inflammasomes are activated by only one or a few highly specific agonists, but NLRP3 can be activated by a variety of unrelated stimuli, including K+ or Cl-, Ca2+, lysosome destruction, mitochondrial dysfunction, metabolic changes, and trans-Golgi disintegration. Depending on the stimulus, NLRP3 inflammasome can be classified into typical caspase-1 activation and atypical caspase-4/5 or caspase-11 (in mice) activation pathways (14).

Here we focus on the canonical activation pathway of NLRP3, which can be mainly divided into two steps. The first is priming, where PAMPs or DAMPs, such as lipopolysaccharide (LPS), are recognized by Toll-like receptors, leading to activation of nuclear factor κB (NF-κB) -mediated signaling pathways (15). NF-κB upregulates the transcription of inflammasome-related components, such as inactive NLRP3, caspase-1, IL-β, and IL-18. This is followed by activation, mainly triggered by ATP, pore-forming toxins, viral RNA, and particulate matter, leading to the assembly of NLRP3, ASC, and pro-caspase-1 into a complex that converts caspase-1, proIL-18, and proIL-1β to their active forms (16).

**Figure 1:**
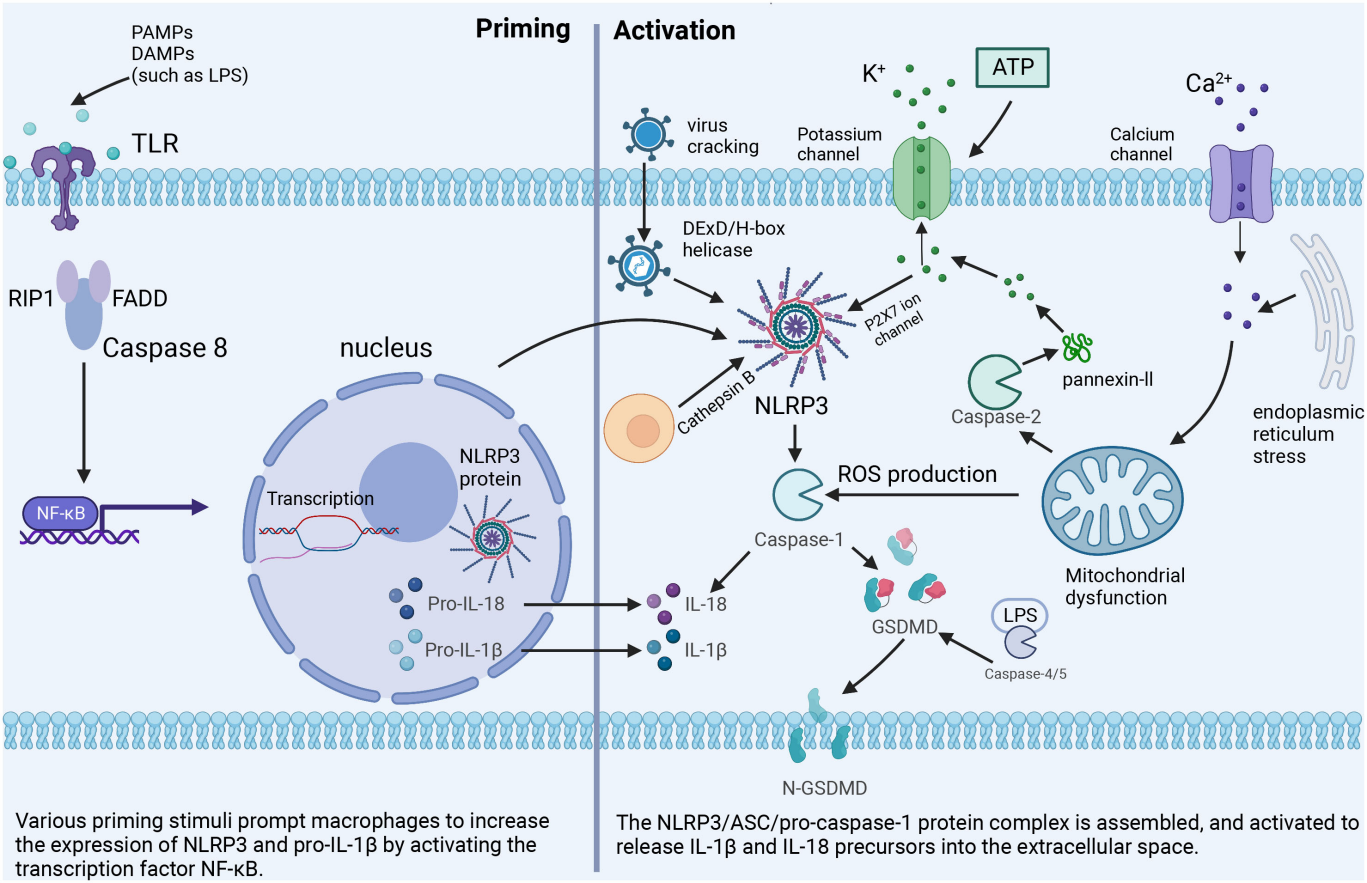
The classical NLRP3 inflammasome activation pathway. Priming: Toll-like receptors are stimulated by PAMPs or DAMPs to up-regulate the expression of NLRP3 inflammasome-related components. Activation: Important cellular signals for inflammasome activation include K+ efflux, Ca2+ flux, ROS, mitochondrial damage, and lysosomal disruption.

Excessive and sustained activation of inflammasome exacerbates the inflammatory response. It induces pyrosis through the specific maturation of IL-1β and IL-18, which is a major pro-inflammatory mechanism and the pathogenesis of various inflammatory disorders (**Figure 1**).

### 2.3 NLRP3 and the development of diabetes mellitus

**Figure 2:**
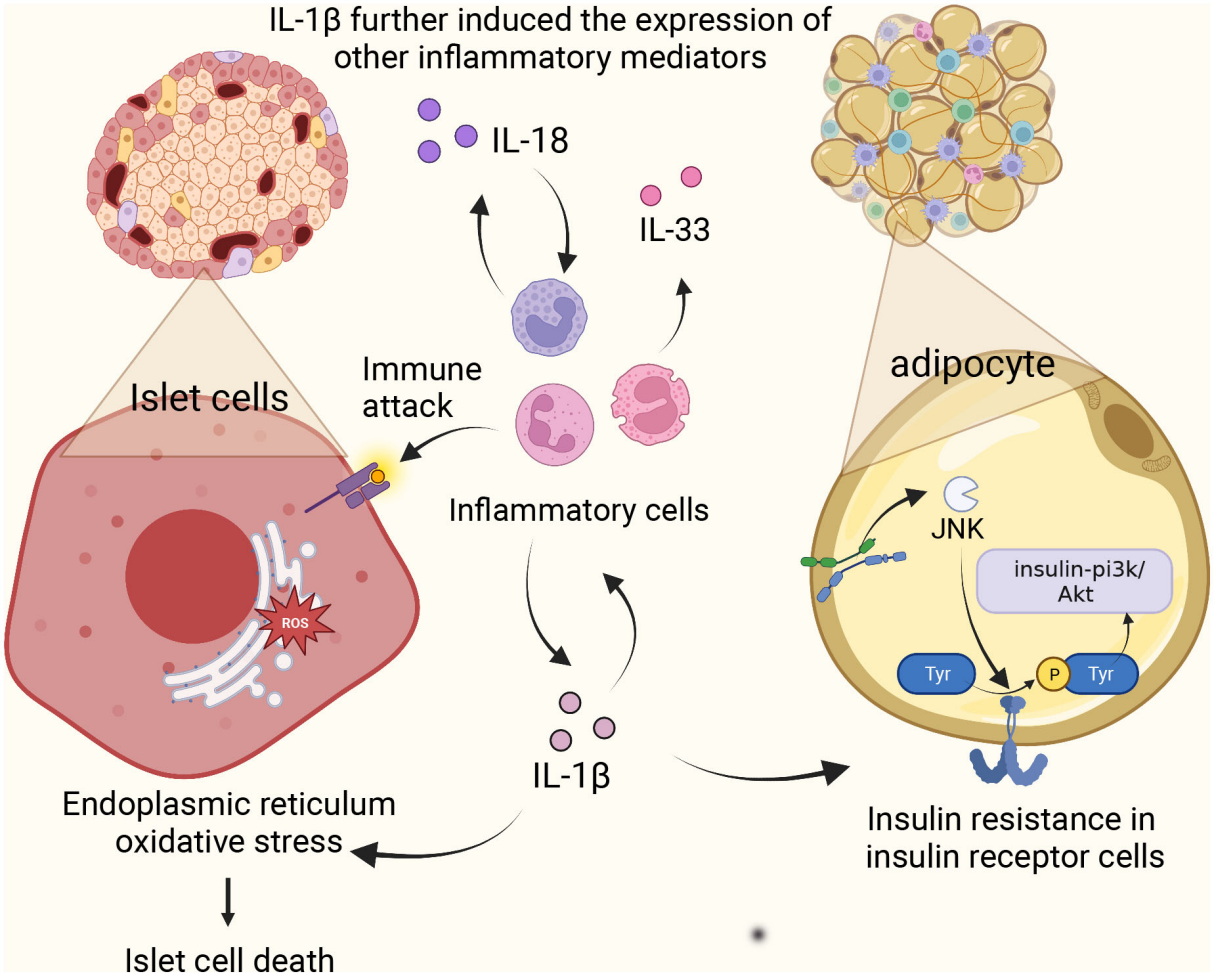
NLRP3 inflammasome complex and pathology in diabetes. Activation of NLRP3 promotes the production of IL-1β and IL-18, amplifies the inflammatory response, causes oxidative stress and endoplasmic reticulum stress, and induces apoptosis of islet beta cells. Meanwhile, high levels of IL-1 induced the activation of c-Jun N-terminal kinase and decreased the activity of insulin-PI3K/Akt signaling pathway. IL-18 induces Th1 over-activation leading to type 1 diabetes.

Activation of NLRP3 inflammasome affects glucose tolerance and insulin sensitivity, i.e., the cellular response of insulin-dependent cells such as adipocytes and cardiomyocytes to the insulin hormone. The function of glucose-induced expression of NCLX, a Na/Ca+2+ exchanger, in rat aortic myoepithelial cells, suggests that NCLX increases Ca2+ flux in glucose-dependent mitochondria, thereby regulating ROS production and subsequent activation of the NLRP3 inflammasome (17). This activation of NLRP3 promotes the production and maturation of IL-1β and IL-18. Overexpression of IL-1β in the body can lead to a variety of consequences. (1) The presence of the IL-1 receptor signal amplifies the inflammatory response and further induces the expression of other inflammatory mediators (IL-18, IL-33) (18), which together with IL-18 induces pyroptosis (19). (2) The overexpression of IL-1β causes oxidative stress and endoplasmic reticulum stress, which leads to the death of human pancreatic epithelial MIA PaCa-2 cells and leads to T2DM (20). (3) High level of IL-1 activates c-Jun N-terminal kinase (JNK). It induces serine phosphorylation of insulin receptor substrate 1 (IRS-1), which further impairs the activity of insulin PI3K/Akt signaling pathway in insulin-sensitive tissues and increases blood glucose. Research has indicated a significant positive correlation between IL-18 and various anthropometric parameters, liver enzymes, fasting and post-load glucose levels, insulin, uric acid, and triglycerides. In contrast, there’s a negative correlation with HDL. To discern individuals with carbohydrate metabolism disorders from those with metabolic syndrome, an ROC analysis was employed to evaluate the circulating levels of IL-18. The AUC for carbohydrate metabolism disorder stood at 0.597 (p = 0.001; 95%CI = 0.539-0.654), while the AUC for metabolic syndrome was 0.581 (p = 0.021; 95%CI = 0.516-0.647). This data suggests that as IL-18 levels rise, carbohydrate tolerance diminishes, potentially leading to the onset of diabetes (21). In addition, the data suggest that carbohydrate tolerance worsens with increasing IL-18 levels (21). Because IL-18 acts as a cofactor for Th1 and Th2 cell development, excessive Th1 activation leads to type 1 diabetes, an autoimmune disease. In addition, it is worth mentioning that researchers have recognized the relationship between IL-18 and IL-1β secretion, which significantly increases the expression of IL-1β (22), suggesting that IL-18 may promote the development of diabetes through its interaction with IL-1β (**Figure 2**).

## 3 Susceptibility links between COVID-19 and diabetes

There may be a certain relationship between COVID-19 infection and hyperglycemia. Infection with COVID-19 can increase blood glucose, aggravate the severity of COVID-19 infection, and make hyperglycemia and even diabetes one of the sequelae of COVID-19. Patients with diabetes are exacerbated by hyperglycemia during infection and recovery.

### 3.1 The prognosis of COVID-19 is hyperglycemia

The study showed slightly higher HbA0c in patients with severe COVID-19 than in those with mild COVID-19.Although this difference was not significant (23), a study based on the glycemic characteristics and clinical findings of hospitalized COVID-19 patients suggests that diabetes and or uncontrolled hyperglycemia frequently occur in hospitalized COVID-19 patients. These COVID-19 patients with diabetes and or uncontrolled hyperglycemia had longer hospital stays and significantly higher mortality than those without diabetes or uncontrolled hyperglycemia, with particularly high mortality among those with uncontrolled hyperglycemia (24).

The reasons for elevated blood glucose in patients with COVID-19 may be as follows. In patients with SARS-CoV-2 infection, C-reactive protein increases sharply. In addition to the increase in blood glucose caused by stress, cytokine storm, a highly inflammatory pathological state caused by a viral infection, can directly and indirectly affect pancreatic β cells and increase blood glucose. Studies have shown that the expression of ACE2 in the pancreas (mainly islet cells) is even higher than that in lung. SARS-CoV-2 enters host cells and circulates in plasma through the ACE2 receptor on the cell surface (25). In addition, some studies have shown that SARS-CoV-2 can disrupt ACE/ACE2 balance and RAAS activation, leading to insulin resistance (26). Most notably, one of the International COVID-19 Inflammation Studies assessed the contribution of inflammation and hyperglycemia to diabetes risk by examining inflammatory biomarkers collected on admission, and blood glucose levels and insulin data throughout the hospital stay. The relationship between diabetes and the prognosis of COVID-19 is mainly mediated by the state of “hyper inflammation” (27). Proinflammatory cytokines and acute-phase reactants such as IL-1β and IL-18 caused by COVID-19 around NLRP3 activation may directly or indirectly cause inflammation and pancreatic β cell damage, resulting in severe insulin resistance and hyperglycemia. These factors further increase the body’s blood glucose, aggravate the inflammatory response, further cause the activation of NLRP3, and finally promote the onset and progression of COVID-19 and diabetes.

### 3.2 Diabetes and susceptibility to COVID-19

Studies have shown that diabetic patients infected with SARS-CoV-2 have higher rates of hospital admissions, severe pneumonia, and mortality than non-diabetic patients infected with SARS-CoV-2. Patients with impaired glucose tolerance or diabetes have been reported to have a 50-60% higher risk of lung infections (28), Monocytes and macrophages are the most abundant immune cell types in the lungs of COVID-19 patients and appear to play a central role in the pathogenicity of the disease (29). These cells adjust their metabolism after infection and become highly glycolytic, which facilitates the replication of SARS-CoV-2 (30). Recent evidence suggests that patients with diabetes are at increased risk for complications of severe adult respiratory distress syndrome and multiple organ failure. Compared to non-diabetic patients, a recent meta-analysis showed that diabetes was associated with a 2.3-fold increased risk of severity and a 2.5-fold increased risk of COVID-19-related death (31). Therefore, it can be concluded that diabetes is a poor prognostic factor for COVID-19.

Patients with diabetes mellitus (DM) are at higher risk of frequent infections than non-diabetic patients, possibly due to some immunodeficiency in diabetic patients. In addition, there is a more complex process in the patient group. Regarding innate immunity of neutrophils, the phagocytosis abilities of chemotaxis, adhesion, phagocytosis, oxidative burst, and killing in diabetic patients are generally lower than in normal people. Therefore, these patients may be more susceptible to COVID-19 virus infection. High blood glucose and insulin resistance can lead to systemic inflammation and activate the NLRP3 pathway, which increases the risk of respiratory infections in diabetes and leads to poor infection treatment outcomes.

Another point of note is that the immediate defense responses to pathogens in patients with COVID-19 are polymorphic, involve macrophages and dendritic antigen-presenting cells, and can be mediated by the humoral system, but these responses are suppressed in diabetes (32), The activation of the NLRP3 pathway can cause a series of cellular immune problems such as inhibition of cytokines, leukocyte recruitment, neutrophil dysfunction, macrophage dysfunction, NK cell dysfunction, inhibition of antibody and complement effects, which is the key to the decline of immunity and increased susceptibility to infection in diabetic patients (33). In addition, this increase in advanced glycation end products in adaptive immunodeficiency can also be associated with impaired production of type 1 interferon by T lymphocytes (34). These causes can lead to a decline in the function of the immune system, ultimately making the patient more susceptible to viral infection.

## 4 Hypoglycemic agents targeted for NLRP3 activation in SARS-CoV-2

Commonly used hypoglycemic drugs are divided into oral hypoglycemic drugs and injection hypoglycemic drugs. Oral hypoglycemic agents can be divided into sulfonylurea secretagogues, biguanides, glienide secretagogues, α-glucosidase inhibitors, thiazolidinediones, DPP-4 inhibitors, SGLT-2 inhibitors. The injectable drugs mainly include insulin. Here, we list some hypoglycemic agents that can act on the NLRP3 inflammasome and explain their mechanism of action. In this paper, we mainly listed oral hypoglycemic drugs, and injectable drugs were represented by GLP-1 receptor agonists. In addition, this paper also listed other drugs that may treat COVID-19 by acting on the NLRP3 inflammasome (**Table 1**).

**Table 1:** The role of NLRP3 inhibitor in the treatment of COVID-19.

Drugs	Drug category	The role and mechanism in the treatment of COVID-19	Model/Object	Reference
Phenformin	biguanides	Metformin reduces the expression of pro-inflammatory cytokines such as TNF-α and IL-6 by blocking LPS-induced ATP-dependent mitochondrial (MT) DNA synthesis and oxidized mtDNA (NLRP3 ligand) production.	pulmonary alveolar macrophage	(35–38)
Sitagliptin	DPP4 inhibitor	DPP4 inhibitor enhanced AMPK phosphorylation and attenuated NLRP3 activation. It may be associated with disease severity in MERS-CoV, but its effect on SARSCoV-2 has not been determined.	Ob/ob mouse model	(39–42)
Empagliflozin	SGLT2 inhibitor	SGLT2 inhibited IL-1β secretion, accompanied by an increase in serum β-hydroxybutyrate (BHB) and a decrease in serum insulin, and inhibited the activation of NLRP3 inflammasome.	Patients with cardiovascular disease and diabetes mellitus	(43–45)
liraglutide	GLP-1 receptor agonist (Injectable drugs)	GLP-1 may alleviate NLRP3 inflammasome-dependent inflammation in PVAT by inhibiting NF-κB signaling.	Diabetic adipose rat model	(46–48)
Pioglitazone	Thiazolidine	RSG can inhibit the activation of NLRP3 inflammasome by activating Nrf2 signaling pathway, and the mechanism may be related to the increased expression of ACE-2.	Mouse model of liver injury	(48–50)
Colchicine	gout suppressant	It is possible by selectively blocking different steps before the oligomerization of NLRP3 inflammasome and by reducing the release of major cytokines (IL-1β and IL-18).	COVID-19 patients	(51, 52)
Hydroxychloroquine	4-aminoquinoline derivatives antimalarial drugs	It combined with artemisinin treatment can inhibit NF-κB signaling and NLRP3 inflammasome activation by inhibiting exosomes in rats.	Rat models with IgA nephropathy	(53)
Pirfenidone	Pyridone	It can inhibit apoptosis, down-regulate ACE receptor expression, reduce inflammation and improve oxidative stress through a variety of mechanisms, thereby protecting the body from cytokine storm.	COVID-19 patients	(54, 55)

### 4.1 The biguanide - metformin (DMBG)

A representative of the biguanide class of hypoglycemic drugs is metformin. Metformin is a first-line drug for the treatment of type 2 diabetes, especially in overweight patients. Metformin activates phosphorylated AMPK, reduces the expression of pro-inflammatory cytokines including TNF-α, IL-6, and IL-1β, and decreases NLRP3 inflammasome activation, thereby suppressing the level of lipopolysaccharides (LPS) and SARS-COV-2-induced ARDS. Studies have shown that metformin blocks LPS-induced ATP-dependent synthesis of mtDNA (an NLRP3 ligand), without relying on the AMP-activated protein kinases AMPK or NF-kB. ComC inhibited AMPK phosphorylation mainly by inducing the release of inflammatory cytokines and increasing the activation of NLRP3 inflammatory bodies, resulting in decreased survival of cardiomyocytes. Metformin exerts cardioprotective effects by modulating the inflammatory response induced by myocardial I/R injury, which largely depends on the enhancement of the AMPK pathway, thereby inhibiting the activation of NLRP3 inflammasome (35). The anti-inflammatory properties of metformin have been demonstrated in various autoimmune inflammation models, such as arthritis, uveitis, and hepatitis (56). In addition, metformin inhibits NLRP3 inflammasome activation and alveolar macrophage production of interleukin-IL-1β, as well as inflammasome independent IL-6 secretion, thereby reducing lipopolysaccharide (LPS) and SARS-CoV-2-induced ARDS. In a US study, metformin was found to reduce deaths from COVID-19 by a factor of 10 in African American patients with type 2 diabetes (57).

In an observational study, when exposed to COVID-19, patients treated with metformin had significantly lower mortality than those not treated with metformin (OR: 0.59, 95% CI: 0.43-0.79; P = 0.001). This suggests that mechanisms beyond glycemic control that underlie the anti-inflammatory activity of metformin could help reduce the risk of severe COVID-19 (58). The COVID-19 disease-specific findings are consistent with the effect of metformin in reducing tumor necrosis factor (TNF-α) levels in women, suggesting that metformin may protect against CCOVID-19 through TNF-α-mediated pathway. This finding has been confirmed by Yuchen Chen, who found the level of IL-6 was reduced in patients treated with metformin (59). Prolonged use of metformin improved other age-related pathologies and extended lifespan and health time in model organisms, and these effects may not be related to glycemic control but to its anti-inflammatory properties (60).

### 4.2 DPP4 inhibitor – daxagliptin

Dipeptidyl peptidase 4 (DPP4) is overexpressed in many adverse environments, such as oxidative stress, inflammation, and apoptosis. It has been reported that there may be a close interaction between COVID-19 peak protein and DPP4. Popular examples of DDP4 inhibitors include saxagliptin (SAX) and vigagliptin (VIL), which can increase endogenous levels of glucagon-like peptide-1 (GLP-1) and glucose-dependent insulin-secreting polypeptide (GIP) by promoting insulin release from pancreatic beta cells. Previous studies have shown that DPP4 inhibitors have anti-inflammatory Reno protective effects in mouse models of type 2 diabetic nephropathy (61), mainly by down-regulating the expression of TNF-α, IL-1β, NLRP3 inflammasome and iNOS, reducing renal tubular injury and protecting renal tissue inflammation (62). Doxorubicin (DXR) induced inflammation and nephrotoxicity are inhibited by several drugs which play an essential role in inhibiting NLRP3 inflammasome (63). Decreased circulating DPP4 activity has been associated with severe COVID-19 disease, the regulation of DPP-4 expression on immune cells induces a wide range of anti-inflammatory and immunomodulatory effects, which can effectively restore the homeostasis of immune response after coronavirus infection and improve the prognosis of patients (64, 65).

### 4.3 SGLT2 inhibitor –dagliflozin

SGLT2 inhibitors are a new type of antiglucose drugs. The representative drug is dagliflozin, which inhibits the reabsorption of glucose in the kidneys. Therefore, excess glucose can be discharged from the urine, thus achieving a lower blood glucose. Meanwhile, SGLT2 inhibitors modulate NLRP3 inflammasome activity through ketones and insulin and lead to a slight increase in serum ketone bodies (β -hydroxybutyrate (BHB)), which not only act as metabolites but also play an important role in cellular signaling. BHB has also been found to inhibit NLRP3 inflammasome activation and reduce IL-1β production in macrophages (66). Moreover, SGLT2 inhibitors are also positively correlated with reducing blood glucose through renal glucose excretion, thereby reducing serum insulin levels. Interestingly, SGLT2 inhibitors also reduce serum uric acid levels by increasing renal clearance of uric acid, a potent activator of NLRP3 inflammatory factors. SGLT2 not only increases serum BHB levels, but also decreases serum insulin, glucose, and uric acid levels, thus inhibiting NLRP3 inflammatory body in general (43).

**Figure 3:**
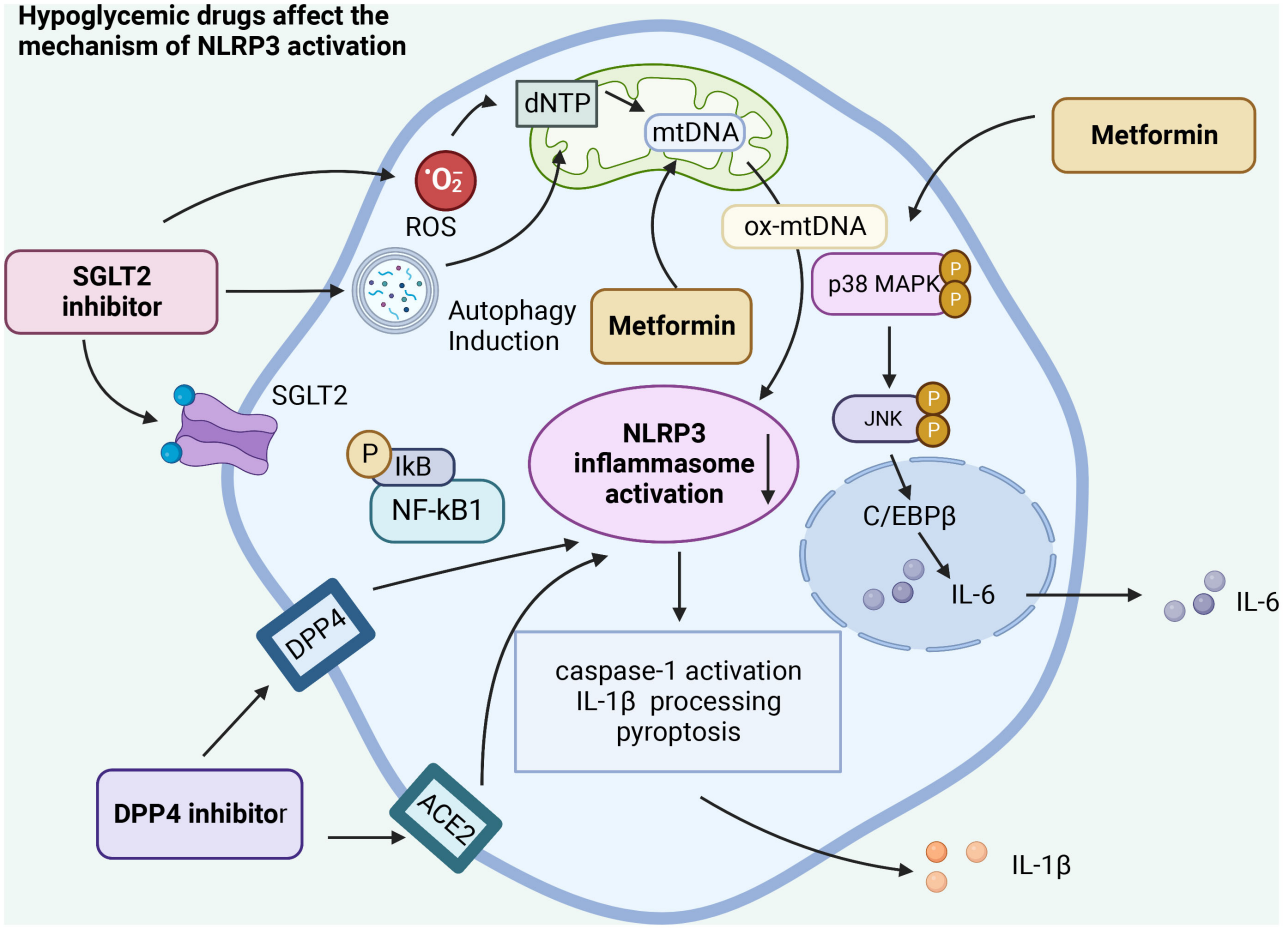
Hypoglycemic agents act on the activation of NLRP3 inflammasome in different ways. Hypo glucose-lowering drugs can affect the activation of NLRP3 inflammasome by acting on different receptors, affecting the autophagy of cells, the release of reactive oxygen species, regulating the NF-κB pathway, and acting on the activation of caspase-1 and the release of IL-1β, ultimately affecting the inflammatory response caused by SARS-CoV-2 infection.

Intriguingly, some studies posit that SGLT2 inhibitors primarily act on inflammatory pathways rather than on pathways associated with glucose regulation. Even under standard glucose conditions, SGLT2 inhibitors can mitigate smooth muscle migration and proliferation. This action is attributed to their impact on oxidative stress, NLRP3 expression, and inflammatory responses in the IL-17α pathway, without inducing cell death (43). Our research suggests that SGLT2 inhibitors influence the NLRP3 inflammasome by diminishing oxidative stress, curbing the NF-kB signaling pathway, and stimulating autophagy. Such mechanisms potentially help suppress inflammation and the cytokine storm disturbances associated with COVID-19 (67). Based on current clinical studies, it can be concluded that SGLT2 has anti-inflammatory properties and can positively affect tissue hypoxia, oxidative stress, autophagy, and energy metabolism, ultimately positively influencing the dysregulation of the cytokine storm of COVID-19 (68) (**Figure 3**).

### 4.4 Thiazolidinediones – rosiglitazone

Thiazolidine is an insulin sensitizer and could reduce blood glucose by increasing peripheral tissue sensitivity to insulin and improving insulin resistance. Rosiglitazone Hydrochloride, a thiazole-pridione insulin sensitizer, is a clinical drug for the treatment of type 2 diabetes mellitus and has the potential to inhibit inflammatory response (49, 69). A basic study found that rosiglitazone may reduce inflammation by inhibiting the expression of NLRP3 inflammatory and tumor necrosis factor-α in macrophages. The results showed that rosiglitazone can reduce the serum levels of IL-1β and tumor necrosis factor-α and reduce the expression of caspase-1 and NLRP3 in inflammatory mouse models, thereby reducing the occurrence of inflammation (70). Clinical studies have shown that rosiglitazone can inhibit NLRP3 inflammasome activation by decreasing caspase-1 and NLRP3 expression and serum IL-1β and TNF-α levels (70). In another randomized controlled trial, insulin resistance and type 2 diabetes were associated with low levels of inflammation, and thiazolidinediones were effective in controlling inflammatory markers and metabolic parameters (71). Pioglitazone is also a common thiazolidinedione glucose-lowering agent. In detail, Pioglitazone ameliorates oxidative stress and inhibits NLPR3 inflammasome activation by activating AMPK signaling and regulating autophagy levels, finally reducing the release of inflammatory cytokines IL-1β and IL-18 (72).

### 4.5 Injectable drugs – GLP-1 glucagon-like peptide

GLP-1 is a novel hypoglycemic drug that can activate the GLP-1 receptor and enhance insulin secretion in a glucose concentration-dependent manner to reduce blood glucose. NLRP3 inflammasome activation promotes the release of inflammatory cytokines such as IL-1β and IL-6 and triggers the production of intestinal GLP-1 (46). According to previous studies, GLP-1 may alleviate NLRP3 inflammasome-dependent inflammation in PVAT by inhibiting NF-κB signaling. In treating GLP-1, clinical studies have demonstrated that GLP-1 exerts anti-inflammatory effects by inhibiting NF-κB, IL-1β, IL-6, TNF-α and NLRP3 inflammatory pathways (73). Currently, some clinical studies have proved that GLP-1 agonists, such as liraglutide, exhibit anti-inflammatory activity by activating adenylate cyclase (AC) to produce cyclic adenosine phosphate (CAMP), and then activating protein kinase A (PKA) to upregulate the CAMP reaction of primary binding protein (CREB). Liraglutide has also been shown to reduce IL-6 levels in patients (74). Another randomized controlled trial also noted that GLP-1, which counteracts oxidative stress, inflammation and endothelial dysfunction caused by changes in blood sugar, can reduce the secretion of soluble intercellular adhesion molecule-1 and interleukin-6 (75).

## 5 Conclusion

Given the status of the global pandemic of COVID-19 virus, although there is insufficient evidence that diabetic patients tend to be more susceptible to infection, the greater severity of disease after infection has already been confirmed in hyperglycemic populations. This review summarizes the mechanisms by which NLRP3 inflammatory vesicles cause inflammatory storms in COVID-19 infection and the chronic low inflammatory state in diabetic patients caused by NLRP3. Moreover, the key role of NLRP3and diabetes in COVID-19 infection is well discussed, and several hypoglycemic agents have been proposed as an adjunctive treatment for diabetic patients infected with COVID-19 regarding NLRP3.Although further clinical trials are needed to address this issue, it is promising to consider the current research progress.

## Author contributions

This article was conceptualized by JPL, PY and JZ. Literature review and original draft preparation was conducted by JYZ, XM and FL. JTL, ZZ, YC, PPY, YY, XL and DZ provided important scientific and clinical feedback and assisted with editing and finalizing the manuscript. All authors reviewed and commented on previous versions of the manuscript, and all authors read and approved the final manuscript. All authors agree to be accountable for the work presented in the manuscript. Authors JYZ and XM contributed equally to this work.
